# Study on the Deterioration Mechanism of Pb on TiO_2_ Oxygen Sensor

**DOI:** 10.3390/mi14010156

**Published:** 2023-01-07

**Authors:** Chao Duan, Lejun Zhang, Zhaoxi Wu, Xu Wang, Meng Meng, Maolin Zhang

**Affiliations:** 1 China Aerospace Components Engineering Center, China Academy of Space Technology, Beijing 100081, China; 2 School of Advanced Materials and Nanotechnology, Xidian University, Xi’an 710071, China

**Keywords:** TiO_2_, oxygen sensor, lead contaminant, surface state

## Abstract

Previous studies have shown that the pollutants in exhaust gas can cause performance deterioration in air-fuel oxygen sensors. Although the content of Pb in fuel oil is as low as 5 mg/L, the effect of long-term Pb accumulation on TiO_2_ oxygen sensors is still unclear. In this paper, the influence mechanism of Pb-containing additives in automobile exhaust gas on the response characteristics of TiO_2_ oxygen sensors was simulated and studied by depositing Pb-containing pollutants on the surface of a TiO_2_ sensitive film. It was found that the accumulation of Pb changed the surface gas adsorption state and reduced the activation energy of TiO_2_, thus affecting the steady-state response voltage and response speed of the TiO_2_-based oxygen sensor.

## 1. Introduction

Although new energy vehicles represented by electric vehicles have received great attention, fuel oil vehicles are still predominant. Exhaust gas pollution from fuel oil vehicles is a global problem [1,2]. The United States, Japan, Europe and China have successively formulated and implemented increasingly stringent emission regulations for vehicles to strictly limit the harmful gases in vehicle exhaust gas. To satisfy these emission standards, the air–fuel ratio (A/F) must be strictly controlled for all fuel oil vehicles. Oxygen sensors (or lambda sensor) play an important role in controlling A/F in the whole system. They feed back the oxygen information in the exhaust gas of the engine to the ECU (electronic control unit) in real time to keep the A/F ratio of the engine in the optimal range and to ensure that the vehicle emissions meet the strict emission regulations [3,4,5].

ZrO_2_ and TiO_2_ are two typical materials that have been successfully applied as oxygen sensors. The main advantages of ZrO_2_ are its large measurement range and good stability; however, it suffers from the problems of large volume, complex structure and high price [6,7]. TiO_2_-type oxygen sensors have the characteristics of small volume, low cost, good stability and fast response, which have been widely studied [8,9,10,11]. Many methods have been successfully used to improve the performance of TiO_2_-based sensors, such as surface modification by Pd [12] and Ag [13] for electron sensitization, surface modification by Pt [14] and Au [15] for chemical sensitization, and donor/acceptor doping [16,17,18]. In addition, TiO_2_ with various morphologies prepared by different methods, such as nanorods [19], nanospheres [20], heterojunctions [21], etc., has also been successfully employed to improve gas-sensing properties [22,23].

**Figure 1:**
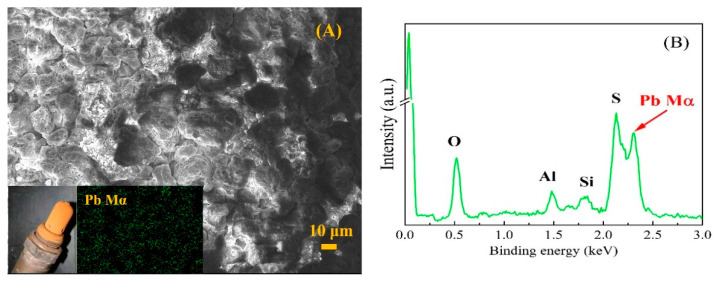
The appearance of a failed sensor (**A**), as well as its surface morphology and Pb distribution (**B**).

Although the additives in fuel oil have been strictly restricted, it is still a common fact that fuel oil contains Pb (5 mg/L), Mn (2 mg/L), S (10 mg/Kg) and other components [24,25]. Therefore, the pollutants such as S, Pb and Mn are inevitably present in the exhaust gas. These contaminants accumulate on the sensor surface for a long time and exert adverse effects [26,27]. Figure 1 shows the surface morphology and energy spectrum data of a failed sensor. It can be seen that a large amount of Pb-containing pollutants (about 3.53 at%) are deposited on the surface.

Binnig et al. [28] confirmed that the presence of S, P, Zn, Mg, K and Ca during the condensation of diesel exhaust gas leads to adverse effects on the PM sensors (particulate matter sensors). They also acknowledged that it is necessary to further study the accumulation of pollutants and the mechanism of chemical action on the surface of sensors. Kornely et al. [29] found that the polarization resistance of YSZ (Yttria-stabilized zirconia) oxygen sensors increased dramatically after being contaminated by Cr, affecting the charge transport. Moos et al. [30] discussed the poisoning mechanism of STF35 (SrTi_0.65_Fe_0.35_O_3_) oxygen sensors in the presence of SO_2_, and proposed a model in which SO_2_ was adsorbed on the surface of STF35 at a low temperature and the SO_2_-STF35 was decomposed into SrSO_4_ and Fe_2_TiO_5_ at a high temperature. In our previous work [31,32,33], the contaminants containing elements such as Mn, P and S were deposited on the surface of TiO_2_ and Pt/TiO_2_ oxygen sensors, respectively, and the mechanism of their influence on the sensor response characteristics was analyzed and discussed.

Earlier, Kocemba et al. reported the performance of Pt/TiO_2_ in pure synthetic exhaust gas and lead-containing (Pb(C_2_H_5_)_4_) exhaust gas [34]. However, there is a lack of clear and thorough research in this area. Moreover, their work mainly focused on the influence of Pb on Pt catalyst, and did not involve the influence of Pb on TiO_2_ sensitive materials. Therefore, TiO_2_ and Pb-contaminated TiO_2_ sensors were designed and fabricated in this paper. Through comprehensive analysis using XRD, SEM and XPS, the influence mechanism of Pb on the gas-sensing characteristics of TiO_2_ air-fuel ratio oxygen sensor was studied in-depth.

## 2. Experiments

TiO_2_ powder was uniformly dispersed in a solution of polyvinyl alcohol and ethyl cellulose to form a slurry. Then, this dispersion was coated onto an Al_2_O_3_ substrate by screen printing. After sintering at 1280 °C for 2 h, a sensitive film was obtained and denoted as TiO_2_.

Pb in fuel oil is mainly derived from tetraethyl lead, which is used as an anti-knock additive in gasoline. However, tetraethyl lead is very volatile and highly toxic at room temperature. Therefore, lead acetate was used as the Pb source in this experiment. To simulate the deposition process of Pb-containing particles in the exhaust gas on the sensor surface, the TiO_2_ film was directly immersed in the lead acetate solution and taken out immediately. After drying in air thoroughly and repeating five times, the treated TiO_2_ thick film was then heat-treated at 800 °C for 2 h and named Pb-TiO_2_.

## 3. Results and Discussion

**Figure 2:**
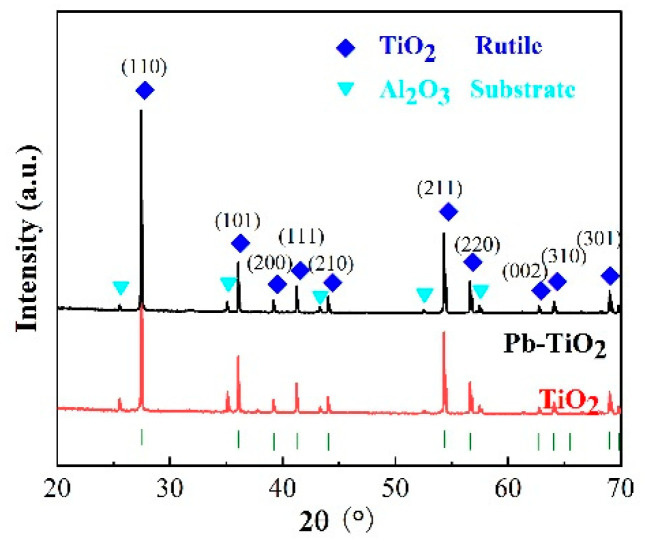
XRD patterns of TiO_2_ and Pb-TiO_2_.

Figure 2 shows the XRD patterns of the TiO_2_ and Pb-TiO_2_ sensitive films. The diffraction peaks of both samples are relatively similar and mainly correspond to TiO_2_ and Al_2_O_3_, with no appearance of obvious impurity peaks. The Al_2_O_3_ peak is derived from the substrate. Due to sintering at the high temperature of 1280 °C, TiO_2_ has a typical rutile structure, which is similar to previous reports [30]. It is worth noting that although the diffraction peaks of both samples are relatively similar, it does not mean that these two samples are completely identical, because of the detection limit of XRD technique.

**Figure 3:**
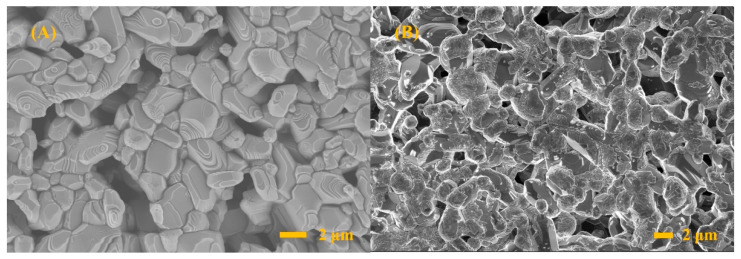

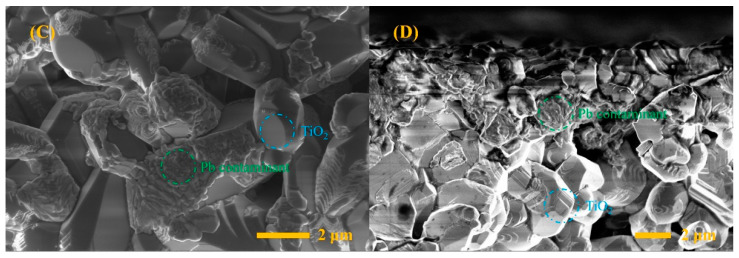
SEM images showing the surface morphology of TiO_2_ (**A**), Pb-TiO_2_ (**B**,**C**) and the cross-sectional morphology of Pb-TiO_2_ (**D**).

Figure 3 presents SEM images of the TiO_2_ and Pb-TiO_2_ sensitive films. It can be seen from Figure 3A that the pure TiO_2_ film has a typical porous structure which facilitates the diffusion and transport of measured gas. The particle size of TiO_2_ is 2–3 μm, and its surface is clean and smooth without any contaminants; this is suitable for the purpose of the experiment. However, the surface morphology of the sample treated with Pb changes significantly. There are obvious contaminants attached to the surface of TiO_2_ particles. The cross-sectional morphology of the thick film shows that the contaminants are mainly attached on the surface of the TiO_2_ film and less inside. This is similar to the situation in which Pb-containing particles in the exhaust gas are deposited on the surface of a sensor’s sensitive film under actual working conditions.

**Figure 4:**
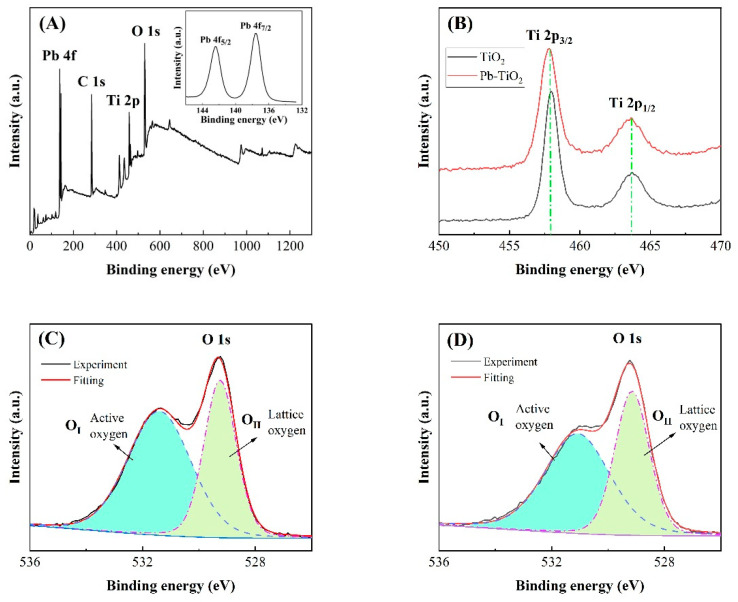
XPS spectra of the surface element states; full spectrum of Pb-TiO_2_. (**A**), Ti2p spectrum (**B**), and O1s spectrum of TiO_2_ (**C**) and Pb-TiO_2_ (**D**).

XPS analysis of the surface chemical states shows the existence of Ti and O elements, as shown in Figure 4. In particular, Pb with content of 3.03 at% is only present in Pb-TiO_2_ sample, which is as expected. Comparing the results before and after Pb contamination, it is found that the characteristic peaks of Ti are almost unchanged, which also indicates that the deposition of Pb has no influence on the chemical state of Ti. It is further found that the O1s peaks overlap, implying that the oxygen on the surface of sample exhibits different chemical states. By dividing the O1s peak, it is found that the O1s peaks of the two samples are composed of two parts. Specifically, the OI peak at 532 eV originates from the adsorbed oxygen on the surface, while the OII peak at 529 eV originates from the lattice oxygen [15,35,36,37]. The proportion of surface adsorbed oxygen is about 60.2% for the TiO_2_ sample, while it is reduced to 48.5% for the Pb-TiO_2_ sample. This means that the proportion of adsorbed oxygen on the surface of the TiO_2_ sensitive film is greatly reduced after Pb treatment. It is well known that the gas sensing properties of metal oxide semiconductors are closely related to the state of oxygen adsorbed on the surface.

**Figure 5:**
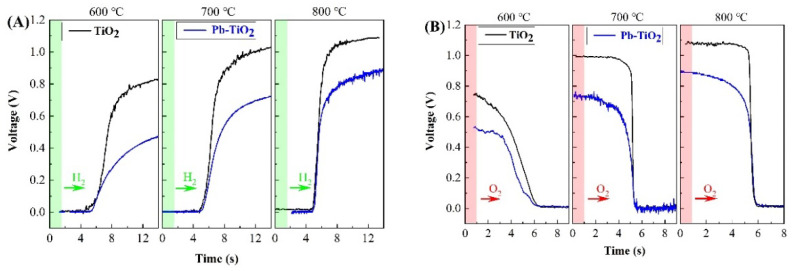
Response characteristics of TiO_2_ and Pb-TiO_2_ in H_2_ (**A**) and O_2_ (**B**) atmosphere.

The dynamic gas-sensing characteristics of pure TiO_2_ and Pb-TiO_2_ oxygen sensors are shown in Figure 5. The dynamic response characteristics of the samples were tested by voltammetry using the test system as described in the literature [38]. The concentrations of H_2_ and O_2_ are both 1000 ppm during the test, and the carrier gas is 99.999% N_2_. TiO_2_ only exhibits obvious sensing properties above 600 °C, which is consistent with the results of Jo et al. [39]. Its steady-state response voltage gradually increases from 0.8 V to 1.1 V (saturated state) with a temperature rise from 600 to 800 °C. At 600, 700 and 800 °C, the response times under H_2_ atmosphere are 560, 480 and 320 ms, respectively, and those under O_2_ atmosphere are 840, 80 and 60 ms, respectively. In contrast, the steady-state response voltage of Pb-TiO_2_ is greatly reduced to 0.52 V at 600 °C, and it needs several seconds to reach a steady state. Usually, the reference voltage for the system to judge the gas state is 0.45 V in the A/F control system [40]. It is extremely easy to cause a misjudgment of the control system in this state. In addition, the response speed of the Pb-TiO_2_ sample is greatly reduced. At 600, 700 and 800 °C, the response times are 1820, 1020 and 380 ms, respectively under H_2_ atmosphere, and 1200, 340 and 220 ms, respectively under O_2_ atmosphere. This can severely affect the control of the A/F ratio.

**Figure 6:**
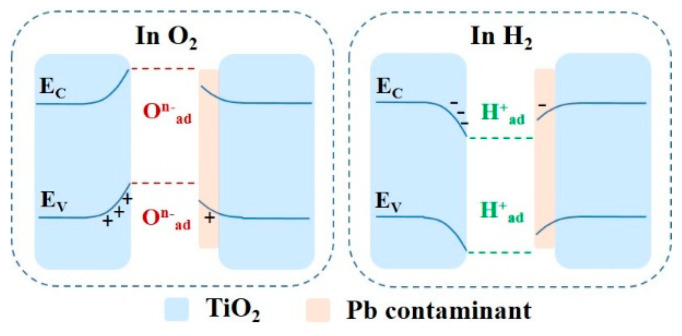
Schematic diagram of the influence mechanism of Pb on the TiO_2_ grain boundary barrier.

After oxygen is adsorbed by TiO_2_ in the air, electrons can be abstracted from the material to form an O^n−^_ad_ acceptor surface state due to the relatively high electron affinity of oxygen [35,36]. As a result, electrons from inside the grains diffuse to the grain boundary interface, causing the accumulation of negative charges on the interface, as shown in Figure 6. This forms a built-in electric field near the interface which hinders the further diffusion of carriers until the accumulation of negative charges on the interface reaches a stable state [9,34]. Reflected in the response characteristics, the resistance increases and the voltage decreases. On the contrary, the electrons released by the reducing gas (H_2_) can lower the charge accumulation on the grain boundary and decrease the additional electrostatic potential energy of electrons near the interface after it adsorbs the reducing gas, which reduces the grain boundary barrier. This is reflected in the response characteristics; both the electric conductance and voltage increase. Obviously, when the Pb contaminants adhere to the surface of TiO_2_, the O^n−^_ad_ content is greatly reduced, resulting in lowering of its grain boundary barrier and thus its sensitivity [41,42]. Peng et al. studied the influence mechanism of Pb on a CeO_2_-WO_3_/TiO_2_-SiO_2_ catalyst. They also found that the introduction of Pb greatly reduced the number of surface acids and redox sites, thereby inhibiting the catalytic performance [43].

The electric resistance value of metal oxide gas-sensing material has the following relationship with the oxygen partial pressure [8]:

(1) $$R=A\exp(\frac{Ea}{KT})Po_{2}^{-\frac{1}{m}}$$

where *A* denotes a constant related to the material, *E*_a_ denotes the activation energy, *K* denotes the Boltzmann constant, *T* denotes the working temperature and *m* denotes a constant that depends on the defect state. Taking the logarithm of both sides of Equation (1), the following expression is obtained:

(2) $$\ln R=\ln A+\frac{E}{kT}-\frac{1}{m}\ln Po_{2}$$

When ln*P_O2_* = 0, taking the corresponding ln*R_1_* and ln*R_2_* at different temperatures *T*_1_ and *T*_2_, the following equation can be obtained:

(3) $$Ea=\frac{k(T_{1}-T_{2})}{T_{1}T_{2}}(\ln R_{1}-\ln R_{2})$$

The *E*_a_ values of TiO_2_ and Pb-TiO_2_ are calculated to be 1.85 and 2.16 eV, respectively. When the measured gases reach the surface of material, they react with the particles adsorbed on the surface of sample. The corresponding reaction rate can be characterized by the Arrhenius formula:

(4) $$r=C\exp(-\frac{Ea}{KT})$$

where *r* denotes the reaction rate and *C* denotes the chemical reaction constant. It can be seen from Equation (4) that the reaction rate of the sensing film decreases with the increase in activation energy, that is, the response time decreases with the decrease in activation energy. Through the calculation of Equation (3), it is found that the *E*_a_ of Pb-TiO_2_ is greatly increased, which also leads to a significant decrease in its response speed.

## 4. Conclusions

In this paper, the influence mechanism of Pb-containing pollutants in exhaust gas on the response characteristics of a TiO_2_ oxygen sensor was studied through simulation. Comparing the dynamic response characteristics of TiO_2_ and Pb-TiO_2_ sensors, it was found that both the steady-state response voltage and response speed of the Pb-TiO_2_ sensor decreased. Together with the results of XRD, SEM and XPS analyses, it was concluded that the deposition of Pb on the surface of TiO_2_ reduced the content of O^n−^_ad_, resulting in a decreasing degree of change in its grain boundary barrier and thus, lower sensitivity. In addition, the surface-deposited Pb reduced the activation energy of TiO_2_, leading to a prolonged response time. Therefore, it is essential to prepare a protective film on TiO_2_ to avoid the direct deposition of Pb-containing particles; this can prolong the working lifetime of TiO_2_-based sensors. Deformable and even foldable materials and devices may be an effective strategy for this protective film [44].

## Data Availability

Not applicable.
